# Medical Items

**Published:** 1916-11

**Authors:** 


### MEDICAL ITEMS

### WHY ATTENTION TO SURGICAL LUBRICATION

"Having devoted especial attention to the treatment of chronic diseases, I think I can attribute a large part of what success I have had to the strict attention I have always given to the matter of elimination. A large percentage of such diseases have as a main factor autointoxication and the retention of morbid waste matters nature fails to expel."

Tongaline by its stimulating action upon the liver, the bowels, the kidneys and the pores is the ideal eliminative and its use will invariably be attended with satisfaction to the physician and the patient.

"The salicylates have long been regarded by the porfession as potent anti-rheumatics, whose efficiency is unapproached by any other known medicine in the treatment of neuralgia, rheumatism, sciatica and other neurotic lesions. Tongaline, from the character of its ingredients, is bound to possess special alterative and eliminative action, with positive affinity for the excretory system of glands, necessarily producing a thorough elimination of the toxic and morbific products of the system through the various emunctories. For rheumatism and gout, sciatica and lumbago, it is the remedy par excellence; its action being absolutely invaluable. In these diseases it thoroughly eiminates the tovaemia which, under other treatments seems every present, hindering convalescence and producing relapses. Tongaline here acts as an efficient alterative and eliminative, removing causes and effecting resolution."

---

### TONICS PREFERABLE TO STIMULANTS.

In selecting a tonic it is highly desirable not to mistake stimulation for tonic action. Stimulation means unduly exciting the higher nerve centers, suddenly and often excessively elevating the blood pressure, and providing a quick but evanescent effect which rapidly passes away and is many times more harmful than beneficial. Tonic action means the gradual restoration of functional efficiency—"helping the body to help itself." Thus, Gray's Glycerine Tonic Comp. is a true tonic, with all the advantages and none of the drawbacks of a stimulant. To state it tersely, "Gray's" is an aid and a support to body functions—a genuine prop—not merely a spur or lash.

---

### A SEDATIVE WHICH MAY BE RELIED UPON.

So many sedative agents have disadvantages of one kind or another that the physician oftentimes is in a quandary as to just what drug or combination to employ. This is particularly so if the patient be a woman or child. In PASADYNE (Daniel's Concentrated Tincture of Passiflora Incarnata), the clinician will find a soporific product which meets every requirement. It not only produces prompt sedation but furthermore is free from disagreeable after-effects. The sleep secured through its administration is tranquil and refreshing. It is especially adapted for use in women and children, for it is free from the dangerous possibilities of other agents so widely employed for the same purpose. Whenever you wish to produce sedation use PASADYNE (Daniel). It has no concern with the Harrison Act. A sample bottle may be had by addressing the Laboratory of John B. Daniel, Inc., Atlanta, Georgia.

---

### OVERCOMING HEPATIC ENGORGEMENT.

Active stimulation of the liver is often urgently needed in certain diseases—notably those of an auto-toxic nature, or characterized by faulty elimination—but not infrequently the efficiency of the remedy used is modified, or completely nullified, by the catharsis incidentally produced. In Chionia, a preparation of Chionanthus Virginica, the practitioner fortunately

IT TAKES THE STING OUT OF PAIN
and yet does not subject the patient to morphine's dangers.
This is what

PASADYNE
(DANIEL'S CONCENTRATED TINCTURE OF PASSIFLORA INCARNATA)

does, and it is the reason why it is being so widely employed as an anodyne. There is no habit-danger attached to PASADYNE (Daniel) but it is effective

ALWAYS THINK OF PASADYNE (Daniel) IN CONGESTION OF THE OVARIES AND IN DYSMENORRHEA, TO MENTION TWO STRIKING INDICATIONS.

*SAMPLES AND LITERATURE SUPPLIED TO PHYSICIANS PAYING EXPRESS CHARGES*

LABORATORY OF JOHN B. DANIEL, INC., ATLANTA, GEORGIA

Holmes Sanatorium

For Surgical & Gynecological Cases
Corner Fourth Avenue
and East First Street
ROME, GEORGIA

CHARTERED TRAINING SCHOOL FOR NURSES.

J. B. S. HOLMES, M. D., Surgeon-in-Chief

Tongaline exerts a manifest action on the nervous system of the secreting order of glands, it diminishes the uric acid content of the blood, and produces a substitutive irritation in the region of the articular surfaces. On account of the exaggerated vasomotor action of Tongaline, the irritation drives the poisonous deposits toward the emunctories, causing a great secretion of bile in the liver, an abundant diuresis in the kidneys and a serious diarrhoea in the intestines, whilei n the feces and in the urine we find a great quantity of uric acid.

These conditions secure the attainment of the desired effect, which is to expel from the organism all those agents, the accumulation and retention of which in the blood are the cause of rheumatism, neuralgia, grippe, gout, nervous headache, malaria, sciatica, lumbago, tonsillitis, heavy colds and excess of uric acid.

has an effective cholagogue that can be relied upon to increase the functional activity of the liver to a marked degree, without unduly stimulating the bowels.

Chionia is invaluable, therefore, for relieving hepatic engorgement, overcoming biliousness and promoting free elimination of the biliary products. In other words, the use of Chionia assures the restoration of hepatic efficiency, but unlike other cholagogues, without catharsis or purgation.

---

### THE COMMONEST OF HUMAN ILLS.

Probably the commonest single ill of modern mankind is what in lay parlance is termed dyspepsia, or in more scientific circles, gastric insufficiency, peptic deficiency, apepsia, and so on ad libitum. The actual condition—the result of abusing the stomach by improper food, irregular feeding, bad habits, etc.—is a marked decline in the secretory activity of the gastric glands. The symptoms are legion, but well summed up by the patient when he speaks of his suffering as ''stomach trouble.''

Recognition of the true state of affairs leaves the physician but one course to follow, activation of the glands of the stomach. Bitter tonics, dilute acids and remedies galore have been used with varying degrees of success, but the remedy that has proven most uniformly satisfactory in restoring functional activity of the gastric glands is Seng. This is a trustworthy tonic to the stomach, a true secerment, that may be relied upon to restore the physiologic activity of the glands and thus overcome the distress and discomfort that make the gastric patient's life so miserable and burdensome. Have you some troublesome case of gastric insufficiency? You will be highly gratified at the results you can obtain with this useful remedy. Write for a sample to Sultan Drug Co., St. Louis, Missouri.

### VAGINAL DISCHARGES.

In many instances vaginal discharges persist for the reason that no energetic local measures are taken to combat the causative condition. Thus, in vaginitis, of either specific or nonspecific origin, if local applications in the form of tampons soaked in an antiseptic solution of positive value were employed in a systematic manner, relief would follow. In this connection the more than ordinary value of ECTHOL (Battle) in vaginal discharges may be mentioned. In vaginitis, used on tampons, it exerts its germicidal influence on the causative organisms and brings about a gratifying relief from the annoying features accompanying the vaginitis. ECTHOL will also be found of much service in cervical erosions.

---

### THE EFFECT OF STIMULATING CELL NUTRITION.

In many conditions of a chronic character improvement may be expected to follow the use of agents calculated to influence nutrition of the individual cells. Thus, if the cells be stimulated to better assimilation and elimination, diseased states due to interference with these normal functions of the cellular constituents of the vital organs must of necessity undergo a change, for the underlying and continuing cause is being altered. The drugs usually employed for this purpose are those termed the alteratives, an efficient representative of which class is IODIA (Battle).

IODIA is a combination of iodide of potash with the active principles of the green roots of stillingia, helonias, saxifraga and menispermum. It has been found in severe clinical tests to exert an influence on the vital functions, the explanation of its favorable effect being sought for in the stimulating action of its several constituents on the normal processes of the body's cells. In chronic gout and rheumatism, glandular diseases and chronic affections of the skin IODIA will offer evidence of its therapeutic value.

### HALF A CENTURY'S PROGRESS.

October 1916 points an epoch in the history of Parke, Davis & Co. The house was founded in 1866—just fifty years ago this month—largely upon the optimism of three or four determined men, backed by a capital that would seem insignificant today. There was nothing in its unpretentious origin to foretell the success of after years. And by success we mean not merely material prosperity, but also that broader and more enduring success that is based upon good-will and confidence.

Manufacturing pharmacy was then a crude, imperfect art. Bacteriology, pharmacology and biological pharmacy were as yet unborn. There were no curative sera or vaccines in those days. Prophylaxis was in its infancy. Standardization was unknown.

Fifty years have wrought marvelous changes in means and methods for the treatment of human ills. The materia medica has been amplified beyond the dreams of the earlier investigators. Knowledge of pathology has immensely broadened. The empiricism of the past has given way to rational therapeutics, the medicine is taking its rightful place among the sciences.

In all these forward movements Parke, Davis & Co. have had some part—notably as discoverers of new vegetable drugs, as inventors of new chemical compounds, as pathfinders and producers in the field of biological manufacture, as investigators in original research, as pioneers in both chemical and physiological standardization.

The past half-century, as we have intimated, has been remarkable in its contributions to the newer materia medica.

### REPRINTS

Of Original Articles published in the Journal-Record of Medicine will be furnished at the following prices:

**500 Copies 50c per page.**

**1000 Copies 75c per page.**

When Reprints are desired it should be indicated on the copy.

SAL HEPATICA

AN EFFERVESCING

SALINE COMBINATION

LAXATIVE AND

ELIMINANT

BRISTOL-MYERS CO.

NEW YORK

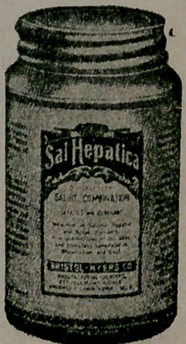

What will the next fifty years bring forward? Time alone can write the answer. Ours is a progressive age. The science of medicine has not reached its highest development. The physician's armamentarium will be further enlarged and fortified. New remedial agents will come into being. Many existing products will be improved. And with the fulfillment of these conditions, Parke, Davis & Co. (if we may judge the future by the past) are certain to be identified.

---

### PALATABLE COD LIVER OIL.

Palatable cod liver oil must be doubly effective because the patient can continue taking it. A generation of physicians have looked upon Cord. Ext. Ol. Morrhuae Comp. (Hagee) as the ideal cod liver oil preparation because of its therapeutic potency and marked palatability. Cord. Ext. Ol. Morrhuae Comp. (Hagee) is particularly useful in chronic nervous exhaustion when the nerve centres are implicated.

---

### THE RANGE OF PASADYNE'S USEFULNESS.

To those who have long employed Pasadyne (Daniel) and are well acquainted with its distinct value in medicine, it will not be fresh information to be assured that Pasadyne (Daniel) has as wide a therapeutic range as any agent of similar character, and with the added advantage of freedom from untoward effects.

In writing of Passiflora Incarnata, and of course, it is scarcely necessary to mention that Pasadyne is merely the distinctive name for Daniel's Concentrated Tincture of Passiflora Incarnata, Potter says that "it has been administered with satisfactory results in neuralgia, chorea, spasmodic asthma, pertussis, hysteria, dysmenorrhea, insomnia, infantile and puerperal convulsions and the opium habit."

A sample bottle may be obtained by addressing the Laboratory of John B. Daniel, Atlanta, Georgia.

